# Association between Class III Obesity (BMI of 40–59 kg/m^2^) and Mortality: A Pooled Analysis of 20 Prospective Studies

**DOI:** 10.1371/journal.pmed.1001673

**Published:** 2014-07-08

**Authors:** Cari M. Kitahara, Alan J. Flint, Amy Berrington de Gonzalez, Leslie Bernstein, Michelle Brotzman, Robert J. MacInnis, Steven C. Moore, Kim Robien, Philip S. Rosenberg, Pramil N. Singh, Elisabete Weiderpass, Hans Olov Adami, Hoda Anton-Culver, Rachel Ballard-Barbash, Julie E. Buring, D. Michal Freedman, Gary E. Fraser, Laura E. Beane Freeman, Susan M. Gapstur, John Michael Gaziano, Graham G. Giles, Niclas Håkansson, Jane A. Hoppin, Frank B. Hu, Karen Koenig, Martha S. Linet, Yikyung Park, Alpa V. Patel, Mark P. Purdue, Catherine Schairer, Howard D. Sesso, Kala Visvanathan, Emily White, Alicja Wolk, Anne Zeleniuch-Jacquotte, Patricia Hartge

**Affiliations:** 1 Division of Cancer Epidemiology and Genetics, National Cancer Institute, Bethesda, Maryland, United States of America; 2 Department of Nutrition, Harvard School of Public Health, Boston, Massachusetts, United States of America; 3 Department of Epidemiology, Harvard School of Public Health, Boston, Massachusetts, United States of America; 4 Division of Cancer Etiology, Department of Population Sciences, Beckman Research Institute, City of Hope, Duarte, California, United States of America; 5 Westat, Rockville, Maryland, United States of America; 6 Cancer Epidemiology Centre, Cancer Council of Victoria, Melbourne, Australia; 7 Centre for Molecular, Environmental, Genetic, and Analytic Epidemiology, University of Melbourne, Melbourne, Australia; 8 Department of Epidemiology and Biostatistics, School of Public Health and Health Services, George Washington University, Washington, District of Columbia, United States of America; 9 Center for Health Research, School of Public Health, Loma Linda University, Loma Linda, California, United States of America; 10 Department of Medical Epidemiology and Biostatistics, Karolinska Institutet, Stockholm, Sweden; 11 Department of Community Medicine, Faculty of Health Sciences, University of Tromsø—The Arctic University of Norway, Tromsø, Norway; 12 Department of Research, Cancer Registry of Norway, Oslo, Norway; 13 Samfundet Folkhälsan, Helsinki, Finland; 14 Department of Epidemiology, School of Medicine, University of California, Irvine, California, United States of America; 15 Division of Cancer Control and Population Sciences, National Cancer Institute, Bethesda, Maryland, United States of America; 16 Divisions of Preventive Medicine and Aging, Brigham and Women's Hospital, Harvard Medical School, Boston, Massachusetts, United States of America; 17 Department of Epidemiology, Biostatistics, and Population Medicine, Loma Linda University School of Public Health, Loma Linda, California, United States of America; 18 Epidemiology Research Program, American Cancer Society, Atlanta, Georgia, United States of America; 19 Massachusetts Veteran's Epidemiology, Research and Information Center, Geriatric Research Education and Clinical Center, VA Boston Healthcare System, Boston, Massachusetts, United States of America; 20 Division of Nutritional Epidemiology, National Institute of Environmental Medicine, Karolinska Institutet, Stockholm, Sweden; 21 Epidemiology Branch, National Institute of Environmental Health Sciences, National Institutes of Health, Department of Health and Human Services, Research Triangle Park, North Carolina, United States of America; 22 Division of Epidemiology, Department of Population Health and NYU Cancer Institute, NYU School of Medicine, New York, New York, United States of America; 23 Johns Hopkins Sidney Kimmel Comprehensive Cancer Center, Baltimore, Maryland, United States of America; 24 Johns Hopkins Bloomberg School of Public Health, Baltimore, Maryland, United States of America; 25 Fred Hutchinson Cancer Research Center, Seattle, Washington, United States of America; University of Cambridge, United Kingdom

## Abstract

**Background:**

The prevalence of class III obesity (body mass index [BMI]≥40 kg/m^2^) has increased dramatically in several countries and currently affects 6% of adults in the US, with uncertain impact on the risks of illness and death. Using data from a large pooled study, we evaluated the risk of death, overall and due to a wide range of causes, and years of life expectancy lost associated with class III obesity.

**Methods and Findings:**

In a pooled analysis of 20 prospective studies from the United States, Sweden, and Australia, we estimated sex- and age-adjusted total and cause-specific mortality rates (deaths per 100,000 persons per year) and multivariable-adjusted hazard ratios for adults, aged 19–83 y at baseline, classified as obese class III (BMI 40.0–59.9 kg/m^2^) compared with those classified as normal weight (BMI 18.5–24.9 kg/m^2^). Participants reporting ever smoking cigarettes or a history of chronic disease (heart disease, cancer, stroke, or emphysema) on baseline questionnaires were excluded. Among 9,564 class III obesity participants, mortality rates were 856.0 in men and 663.0 in women during the study period (1976–2009). Among 304,011 normal-weight participants, rates were 346.7 and 280.5 in men and women, respectively. Deaths from heart disease contributed largely to the excess rates in the class III obesity group (rate differences = 238.9 and 132.8 in men and women, respectively), followed by deaths from cancer (rate differences = 36.7 and 62.3 in men and women, respectively) and diabetes (rate differences = 51.2 and 29.2 in men and women, respectively). Within the class III obesity range, multivariable-adjusted hazard ratios for total deaths and deaths due to heart disease, cancer, diabetes, nephritis/nephrotic syndrome/nephrosis, chronic lower respiratory disease, and influenza/pneumonia increased with increasing BMI. Compared with normal-weight BMI, a BMI of 40–44.9, 45–49.9, 50–54.9, and 55–59.9 kg/m^2^ was associated with an estimated 6.5 (95% CI: 5.7–7.3), 8.9 (95% CI: 7.4–10.4), 9.8 (95% CI: 7.4–12.2), and 13.7 (95% CI: 10.5–16.9) y of life lost. A limitation was that BMI was mainly ascertained by self-report.

**Conclusions:**

Class III obesity is associated with substantially elevated rates of total mortality, with most of the excess deaths due to heart disease, cancer, and diabetes, and major reductions in life expectancy compared with normal weight.

*Please see later in the article for the Editors' Summary*

## Abstract 2

In a pooled analysis of 20 prospective studies, Cari Kitahara and colleagues find that class III obesity (BMI of 40–59) is associated with excess rates of total mortality, particularly due to heart disease, cancer, and diabetes.

*Please see later in the article for the Editors' Summary*

## Editors' Summary

**Background:**

The number of obese people (individuals with an excessive amount of body fat) is increasing rapidly in many countries. Worldwide, according to the Global Burden of Disease Study 2013, more than a third of all adults are now overweight or obese. Obesity is defined as having a body mass index (BMI, an indicator of body fat calculated by dividing a person's weight in kilograms by their height in meters squared) of more than 30 kg/m^2^ (a 183-cm [6-ft] tall man who weighs more than 100 kg [221 lbs] is obese). Compared to people with a healthy weight (a BMI between 18.5 and 24.9 kg/m^2^), overweight and obese individuals (who have a BMI between 25.0 and 29.9 kg/m^2^ and a BMI of 30 kg/m^2^ or more, respectively) have an increased risk of developing diabetes, heart disease, stroke, and some cancers, and tend to die younger. Because people become unhealthily fat by consuming food and drink that contains more energy (kilocalories) than they need for their daily activities, obesity can be prevented or treated by eating less food and by increasing physical activity.

**Why Was This Study Done?:**

Class III obesity (extreme, or morbid, obesity), which is defined as a BMI of more than 40 kg/m^2^, is emerging as a major public health problem in several high-income countries. In the US, for example, 6% of adults are now morbidly obese. Because extreme obesity used to be relatively uncommon, little is known about the burden of disease, including total and cause-specific mortality (death) rates, among individuals with class III obesity. Before we can prevent and treat class III obesity effectively, we need a better understanding of the health risks associated with this condition. In this pooled analysis of prospective cohort studies, the researchers evaluate the risk of total and cause-specific death and the years of life lost associated with class III obesity. A pooled analysis analyzes the data from several studies as if the data came from one large study; prospective cohort studies record the characteristics of a group of participants at baseline and follow them to see which individuals develop a specific condition.

**What Did the Researchers Do and Find?:**

The researchers included 20 prospective (mainly US) cohort studies from the National Cancer Institute Cohort Consortium (a partnership that studies cancer by undertaking large-scale collaborations) in their pooled analysis. After excluding individuals who had ever smoked and people with a history of chronic disease, the analysis included 9,564 adults who were classified as class III obese based on self-reported height and weight at baseline and 304,011 normal-weight adults. Among the participants with class III obesity, mortality rates (deaths per 100,000 persons per year) during the 30-year study period were 856.0 and 663.0 in men and women, respectively, whereas the mortality rates among normal-weight men and women were 346.7 and 280.5, respectively. Heart disease was the major contributor to the excess death rate among individuals with class III obesity, followed by cancer and diabetes. Statistical analyses of the pooled data indicate that the risk of all-cause death and death due to heart disease, cancer, diabetes, and several other diseases increased with increasing BMI. Finally, compared with having a normal weight, having a BMI between 40 and 59 kg/m^2^ resulted in an estimated loss of 6.5 to 13.7 years of life.

**What Do These Findings Mean?:**

These findings indicate that class III obesity is associated with a substantially increased rate of death. Notably, this death rate increase is similar to the increase associated with smoking among normal-weight people. The findings also suggest that heart disease, cancer, and diabetes are responsible for most of the excess deaths among people with class III obesity and that having class III obesity results in major reductions in life expectancy. Importantly, the number of years of life lost continues to increase for BMI values above 50 kg/m^2^, and beyond this point, the loss of life expectancy exceeds that associated with smoking among normal-weight people. The accuracy of these findings is limited by the use of self-reported height and weight measurements to calculate BMI and by the use of BMI as the sole measure of obesity. Moreover, these findings may not be generalizable to all populations. Nevertheless, these findings highlight the need to develop more effective interventions to combat the growing public health problem of class III obesity.

**Additional Information:**

Please access these websites via the online version of this summary at http://dx.doi.org/10.1371/journal.pmed.1001673.

- The US Centers for Disease Control and Prevention provides information on all aspects of overweight and obesity (in English and Spanish)
- The World Health Organization provides information on obesity (in several languages); Malri's story describes the health risks faced by an obese child
- The UK National Health Service Choices website provides information about obesity, including a personal story about losing weight
- The Global Burden of Disease Study website provides the latest details about global obesity trends
- The US Department of Agriculture's ChooseMyPlate.gov website provides a personal healthy eating plan; the Weight-Control Information Network is an information service provided for the general public and health professionals by the US National Institute of Diabetes and Digestive and Kidney Diseases (in English and Spanish)
- MedlinePlus provides links to other sources of information on obesity (in English and Spanish)

## Introduction

Obesity (body mass index [BMI]≥30 kg/m^2^) increases the risk for numerous adverse health outcomes, including most major chronic diseases [1]–[3]. Class III, or extreme, obesity (BMI≥40 kg/m^2^) [4] is emerging as a major public health problem in several developed countries [5]–[7], most notably in the US, where BMI>30, 40, or 50 kg/m^2^ among adults has increased more than 2-, 4-, and 10-fold, respectively, since the mid-1980s [8]. While class III obesity currently affects 6% of the US adult population [8],[9], those belonging to this group accounted for 20% of the total per capita health-care expenditures in 2000 [10].

Effective interventions and accurate projections of future health-care costs requires a better understanding of the health risks associated with class III obesity, but the necessary data are scarce. Because class III obesity was relatively uncommon in the recent past, the few studies that have specifically evaluated mortality rates associated with class III obesity generally had limited sample sizes (e.g., fewer than 400 deaths [2],[11]) or did not separately evaluate risks of death for BMI of 50 kg/m^2^ or higher [2],[3]. As such, there is little quantitative information about the burden of disease, including total and cause-specific mortality rates, for individuals with BMI values of 40 kg/m^2^ and above.

We combined original data from 20 prospective studies from the United States, Sweden, and Australia to evaluate—in what is to our knowledge the largest study on this topic to date—the excess rates of death overall and due to a wide range of specific causes, as well as the expected number of years of life lost attributable to class III obesity.

## Methods

### Study Population

The study sample included participants from the 20 cohort studies in the National Cancer Institute Cohort Consortium that met the eligibility criteria (>5 y of follow-up, >1,000 deaths among non-Hispanic white participants, baseline year 1970 or later) and had the ability to submit data for a previous pooled analysis of BMI and mortality [3], including the Adventist Health Study–I [12], Agricultural Health Study [13], Breast Cancer Detection Demonstration Project [14], California Teachers Study [15], Cancer Prevention Study–II [16], CLUE–I and –II [17], Cohort of Swedish Men [18], Health Professionals Follow-Up Study [19], Iowa Women's Health Study [20], Melbourne Collaborative Cohort Study [21], New York University Women's Health Study [22], National Institutes of Health–American Association of Retired Persons Diet and Health Study (NIH-AARP) [23], Nurses' Health Study–I [24], Physicians' Health Study–I and –II [25], Prostate, Lung, Colorectal and Ovarian Cancer Screening Trial [26], Swedish Mammography Cohort [27], Swedish Women's Lifestyle and Health Study [28], United States Radiologic Technologists Study [29], VITamins and Lifestyle Study [30], and the Women's Health Study [31]. Baseline was defined as the date of completion of the first questionnaire that collected information on key variables (e.g., height, weight, smoking, and personal history of chronic diseases). Personal history of chronic diseases and other important covariates, including race/ethnicity, education, marital status, alcohol consumption, and physical activity level, were self-reported. Height and weight information was self-reported in all but one cohort [21]. Individual-level data were formatted uniformly across studies and combined.

Participants who were younger than 18 y or older than 85 y old at baseline, participants with less than 1 y of follow-up, and participants with BMI≥60 kg/m^2^ were excluded. We also excluded participants who reported having ever smoked cigarettes and/or a history of heart disease, cancer, stroke, or emphysema, as tobacco use and these preexisting illnesses have been demonstrated to confound the association between BMI and mortality [3]. The final sample consisted of 9,564 individuals with a BMI of 40.0 to 59.9 kg/m^2^ (1,575 men and 7,989 women) and 304,011 with a BMI of 18.5 to 24.9 kg/m^2^ (75,680 men and 228,331 women).

### Follow-Up

Participants were followed from the date of completion of the baseline questionnaire to death, loss to follow-up, or administrative end date, whichever occurred first. Causes of death were ascertained from death records or registries and coded according to the Surveillance, Epidemiology, and End Results Program recodes [32] based on cause of death codes defined by the International Classification of Diseases, eighth, ninth, or tenth revisions [33]–[35], and categorized according to the 2002 National Vital Statistics Report of the US Centers for Disease Control and Prevention [36].

### Statistical Methods

We calculated age-adjusted mortality rates (number of deaths per 100,000 persons per year) for the class III obesity (BMI 40.0–59.9 kg/m^2^) and normal-weight (BMI 18.5–24.9 kg/m^2^) groups using direct standardization for age [37], in which rates were weighted according to the age distribution of the total US population in 2000, restricted to ages 20–84 y, using 5-y categories of attained age. We used the US standard to most closely approximate the age distributions of the countries from which the study populations were selected. Specific causes of death were chosen from among the top leading causes of death in the US population in 2000 [36], including (but not limited to) heart disease, cerebrovascular disease, malignant neoplasms, chronic lower respiratory diseases, accidents, diabetes mellitus, influenza and pneumonia, kidney disease (nephritis, nephrotic syndrome, and nephrosis), septicemia, and chronic liver disease and cirrhosis. Mortality rates from suicide and homicide and from major causes of death resulting in fewer than five events in both men and women with BMI values of 40.0 kg/m^2^ and above (e.g., Alzheimer disease) are not shown. Within the major categories of heart disease, malignant neoplasms, and cerebrovascular disease, where the sample size was sufficient, we additionally calculated mortality rates due to more specific causes (e.g., ischemic heart disease). Rate differences were calculated as the difference in age-adjusted mortality rates between the two BMI groups, and two-sided *p*-values for these differences were calculated using a two-sample *Z* test with the variance calculated under the assumption that the number of deaths in each group followed an independent Poisson distribution.

We used proportional hazards regression models, with attained age as the underlying time metric, to calculate hazard ratios (HRs) and 95% confidence intervals (CIs) for total mortality and mortality due to major causes in relation to class III obesity (BMI 40.0–59.9 kg/m^2^) compared with normal weight (BMI 18.5–24.9 kg/m^2^). All models were adjusted for sex, race/ethnicity, education, alcohol intake, level of physical activity, and study. In separate models, we additionally adjusted for self-reported history of diabetes mellitus and hypertension to evaluate the potential for mediation by these co-morbid conditions. In separate models restricted to participants with BMI values between 40.0 and 59.9 kg/m^2^, BMI was included as a continuous variable to evaluate HRs and 95% CIs per 5 kg/m^2^ BMI and to calculate tests for trend. We observed no evidence of violation of the proportional hazards assumption based on graphical assessment of cumulative hazards plots by BMI category.

To calculate years of life lost, we calculated direct adjusted survival curves for each BMI category [38],[39]. Proportional hazards regression models were used to estimate probabilities of survival at each age for each individual, which were then averaged to obtain an overall summary curve. Curves for each BMI category were estimated by counterfactual (i.e., applying the beta for each BMI category in turn to estimate individual and overall survival curves). Years of life lost were calculated as the difference between the adjusted median life expectancy for a given BMI category and the reference category of BMI 18.5–24.9 kg/m^2^. Models were restricted to participants who were 40 y and older at baseline.

As a secondary analysis and alternative approach to combining results across studies, we additionally calculated pooled HRs for class III obesity and total mortality using a random effects meta-analysis model. Heterogeneity between studies was calculated using the *I* ^2^ index [40]. Confidence intervals for the *I* ^2^ index were calculated according to the method described by Thorlund et al. [41]. We conducted subgroup analyses within the aggregate dataset to evaluate potential sources of between-study heterogeneity. For instance, because the association between BMI and mortality has been shown to differ by age at BMI measurement/report and sex [3], and medical care for extremely obese individuals may have improved over calendar time, we calculated HRs and 95% CIs within strata of these factors. We also evaluated geographic location (US versus non-US cohorts) as another potential source of between-study heterogeneity. Tests for interaction by sex, geographic location (US versus non-US cohorts), attained age, and calendar period of follow-up (before and after age 65 y, and before and after the year 2000) were conducted by including a cross-product term in the model. Models evaluating interactions by attained age and calendar period were time-dependent, in which person-time for each participant was split before and after age 65 y or the year 2000.

## Results

A description of participants with BMI in the class III obesity range from each of the 20 cohorts is shown in Table 1. In total, 1,036 of the 9,564 participants died during the follow-up period (median = 9 y, maximum = 27 y).

**Table 1:** Description of participants with BMI in the normal-weight (18.5–24.9 kg/m^2^) and class III obesity (40.0–59.0 kg/m^2^) range, by cohort.

Cohort	Cohort Acronym	Geographic Location	Overall		Class III Obese		Percent Males	Period of Follow-Up	Baseline Age (in Years), Median (Range)	Follow-Up (in Years), Median (Maximum)
			Number of Participants	Number of Deaths	Number of Participants	Number of Deaths				
Adventist Health Study–I [12]	AHS-I	US	11,904	934	128	19	30.9%	1976–1998	51 (26–77)	12 (22)
Agricultural Health Study [13]	AgHealth	US	14,197	312	390	19	36.5%	1993–2008	44 (19–77)	10 (14)
Breast Cancer Detection Demonstration Project [14]	BCDDP	US	9,127	1,296	236	43	0%	1987–2005	62 (48–83)	3 (19)
California Teachers Study [15]	CTS	US	39,541	649	798	21	0%	1995–2005	45 (22–82)	9 (9)
Cancer Prevention Study–II [16]	CPS-II	US	20,989	1,953	488	69	26.2%	1997–2008	63 (45–79)	11 (11)
CLUE–I—Campaign Against Cancer and Stroke; CLUE–II—Cancer and Heart Disease [17]	CLUE	US	4,674	504	151	48	26.0%	1989–2008	45 (24–78)	14 (19)
Cohort of Swedish Men [18]	COSM	Sweden	5,266	303	15	1	100%	1998–2008	62 (45–77)	10 (10)
Health Professionals Follow-Up Study [19]	HPFS	US	8,279	870	38	14	100%	1986–2009	53 (39–71)	17 (22)
Iowa Women's Health Study [20]	IWHS	US	10,222	1,890	329	118	0%	1986–2005	60 (55–70)	19 (19)
Melbourne Collaborative Cohort Study [21]	MCCS	Australia	8,060	481	270	33	24.9%	1990–2008	55 (40–72)	15 (18)
New York University Women's Health Study [22]	NYUWHS	US	3,249	233	48	8	0%	1985–2006	49 (34–68)	19 (20)
National Institutes of Health–American Association of Retired Persons Diet and Health Study [23]	NIH-AARP	US	61,121	3,054	3,069	329	41.7%	1995–2005	60 (50–70)	10 (11)
Nurses' Health Study–I [24]	NHS-I	US	23,450	2,355	368	129	0%	1976–2004	43 (29–55)	26 (27)
Physicians' Health Study–I and –II [25]	PHS	US	7,835	879	19	2	100%	1981–2007	53 (40–57)	23 (26)
Prostate, Lung, Colorectal and Ovarian Cancer Screening Trial [26]	PLCO	US	22,010	1,147	1,280	124	30.0%	1993–2006	60 (55–74)	9 (13)
Swedish Mammography Cohort [27]	SMC	Sweden	6,850	375	27	1	0%	1998–2008	60 (49–77)	10 (10)
Swedish Women's Lifestyle and Health Study [28]	WLH	Sweden	12,838	155	52	2	0%	1991–2006	39 (31–50)	15 (15)
United States Radiologic Technologists Study [29]	USRT	US	22,872	142	742	3	11.6%	1994–2000	45 (34–82)	6 (7)
VITamins and Lifestyle Study [30]	VITAL	US	10,975	206	737	27	32.3%	2000–2007	56 (50–76)	6 (7)
Women's Health Study [31]	WHS	US	10,116	345	379	26	0%	1993–2008	50 (45–73)	13 (15)
**Total**			**313,575**	**18,083**	**9,564**	**1,036**	**24.6%**	**1976–2009**	**57 (19–83)**	**10 (27)**

Compared with participants in the normal-weight range at baseline, those in the class III obesity range included a higher proportion of women, more individuals with baseline age 50 to 69 y (as opposed to younger [19–49 y] or older [70–83 y] individuals), fewer non-Hispanic white individuals, fewer individuals with a college education, more individuals with lower alcohol intake, and more individuals with lower levels of physical activity (Table 2). Among participants within the range of class III obesity, we observed little variation in baseline age, sex, education, and alcohol intake. By contrast, the prevalence of non-Hispanic black race/ethnicity, low physical activity, and history of diabetes, as well as median waist circumference, was generally higher for the top BMI categories.

**Table 2:** Baseline characteristics of participants in the pooled dataset by BMI category.

Characteristic	BMI (kg/m^2^)				
	18.5–24.9 (*n* = 304,011)	40.0–44.9 (*n* = 6,803)	45.0–49.9 (*n* = 1,978)	50.0–54.9 (*n* = 627)	55.0–59.9 (*n* = 156)
**Age**					
19–49 y	35%	23%	23%	21%	17%
50–59 y	27%	40%	40%	48%	44%
60–69 y	29%	33%	33%	27%	33%
70–83 y	9%	4%	4%	4%	6%
**Sex**					
Male	25%	18%	14%	13%	19%
Female	75%	82%	86%	87%	81%
**Race/ethnicity**					
White, non-Hispanic or unknown if Hispanic	93%	90%	88%	87%	85%
Black, non-Hispanic or unknown if Hispanic	1%	6%	8%	8%	10%
Hispanic	2%	2%	2%	2%	3%
Asian/Pacific Islander	3%	<1%	<1%	<1%	1%
Other/unknown	1%	2%	2%	1%	1%
**Education**					
High school graduate or less	22%	31%	32%	26%	31%
Post–high school training/some college	21%	31%	31%	32%	38%
College graduate	54%	36%	33%	39%	28%
Unknown	3%	3%	4%	3%	3%
**Alcohol intake**					
None	32%	45%	47%	48%	46%
>0 to <10 g of ethanol/day	44%	39%	36%	37%	37%
≥10 g of ethanol/day	15%	5%	5%	5%	6%
Unknown	9%	10%	13%	11%	12%
**Physical activity level (cohort-specific tertiles)**					
Low	21%	42%	44%	48%	53%
Medium	24%	20%	17%	15%	12%
High	32%	15%	14%	13%	14%
Unknown	23%	22%	26%	24%	22%
**Waist circumference in cm (males), median** a	89	130	140	141	152
**Waist circumference in cm (females), median** a	74	110	117	122	122
**Prevalent conditions** a					
Diabetes	2%	15%	17%	22%	22%
Hypertension	8%	27%	27%	24%	29%

Data are percent, unless otherwise indicated.

a Among those without missing values. Proportion missing: waist circumference, 73%; diabetes, 8%; hypertension, 32%.

Table 3 compares total and cause-specific mortality rates (deaths per 100,000 persons per year) for the class III obesity and normal-weight groups separately by sex. Total mortality rates for class III obesity participants were 856.0 in men and 663.0 in women. The differences in mortality rates for the two BMI groups were 509.3 and 382.5 in men and women, respectively. Heart disease was the most common underlying cause of death for the class III obesity group (mortality rate differences were 238.9 and 132.8 in men and women, respectively), followed by malignant neoplasms (mortality rate differences were 36.7 and 62.3 in men and women, respectively), and diabetes (mortality rate differences were 51.2 and 29.2 in men and women, respectively). Higher rates of death were also observed for nearly every other major cause of death that we examined, apart from cerebrovascular disease in men and malignant neoplasms of respiratory and intrathoracic organs and diseases of the arteries, arterioles, and capillaries in women, although not all of these differences were statistically significant because of the relatively small numbers of deaths in the class III obesity group for some of the major causes (e.g., malignant neoplasms, chronic lower respiratory disease, and nephritis/nephrotic syndrome/nephrosis deaths in men). The negative rate differences that were observed were also based on small numbers of deaths in the class III obesity group (five, eight, and five deaths due to cerebrovascular disease, malignant neoplasms of respiratory and intrathoracic organs, and diseases of the arteries, arterioles, and capillaries, respectively).

**Table 3:** Age-adjusted cause-specific mortality rates (number of deaths per 100,000 persons per year) by BMI category.

Cause of Death	ICD-10 Code	Men			Women		
		BMI 18.5–24.9 kg/m^2^	BMI 40.0–59.9 kg/m^2^	Difference	BMI 18.5–24.9 kg/m^2^	BMI 40.0–59.9 kg/m^2^	Difference
**Total deaths**		346.7	856.0	509.3**	280.5	663.0	382.5**
**Septicemia**	A40–A41	—	—	—	2.4	8.6	6.1
**Malignant neoplasms**	C00–C97	135.1	171.8	36.7	112.0	174.3	62.3**
Malignant neoplasms of the colon/rectum	C18–C21	15.6	31.1	15.5	13.2	24.8	11.6
Malignant neoplasms of other digestive organs	C15–C17, C22–C26	24.0	31.4	7.3	17.4	19.9	2.5
Malignant neoplasms of respiratory and intrathoracic organs	C32–C34	—	—	—	9.4	3.6	−5.8
Malignant neoplasms of the breast	C50	—	—	—	18.7	30.9	12.2
Malignant neoplasms of the female genital organs	C53–C56	n/a	n/a	n/a	15.4	30.7	15.3**
Malignant neoplasms of the prostate	C61	19.1	20.8	1.7	n/a	n/a	n/a
Leukemia	C91–C95	—	—	—	4.7	13.8	9.0
**Neoplasms (in situ, benign, and of uncertain/unknown behavior)**	D00–D48	—	—	—	2.9	14.7	11.8
**Diabetes mellitus**	E10–E14	5.3	56.5	51.2*	4.3	33.5	29.2**
**Overweight and obesity**	E66	0.0	38.3	38.3*	0.0	20.6	20.6**
**Heart disease**	I00–I09, I11, I13, I20–I51	98.3	337.2	238.9**	112.0	244.8	132.8**
Hypertensive heart disease or heart and renal disease	I11, I13	—	—	—	2.5	16.4	13.9*
Ischemic heart disease	I20–I25	72.2	166.1	93.9**	51.0	147.7	96.7**
Other heart diseases	I26–I51	23.1	150.1	127.0**	19.5	76.6	57.1**
**Essential (primary) hypertension and hypertensive renal disease**	I10, I12	—	—	—	3.3	4.1	0.8
**Cerebrovascular disease**	I60–I69	33.1	22.4	−10.7	33.1	47.2	14.2
Subarachnoid hemorrhage	I60	—	—	—	2.9	4.3	1.4
Intracerebral and other intracranial hemorrhage	I61–I62	—	—	—	6.9	8.7	1.9
**Diseases of the arteries, arterioles, and capillaries**	I70–I78	—	—	—	6.7	5.8	−0.9
**Other disorders of the circulatory system**	I80–I99	—	—	—	0.5	1.5	0.9
**Influenza and pneumonia**	J10–J18	14.9	24.9	10.0	10.4	11.6	1.1
**Chronic lower respiratory disease**	J40–J47	4.3	32.4	28.1	5.5	8.9	3.4
**Chronic liver disease and cirrhosis**	K70, K73–K74	1.6	12.1	10.6*	1.8	7.2	5.4*
**Nephritis, nephrotic syndrome, and nephrosis**	N00–N07,N17–N19, N25–N27	3.6	29.3	25.8	2.7	20.7	18.0**
**Other diseases of the urinary system**	N10–N15, N20–N23, N28–N39	—	—	—	2.9	10.8	8.0
**Accidents**	V01–X59, X85–Y86	—	—	—	13.7	22.9	9.2

Mortality rates were age-standardized using the age distribution of adults aged 20–84 y in the 2000 US census population; not shown if calculations based on fewer than five deaths in the BMI 40.0–59.9 kg/m^2^ group.

*p<0.05;

** *p*<0.001.

ICD-10, International Classification of Diseases, tenth revision; n/a, not applicable.

As an internal comparison, in the subset of participants in the pooled dataset without heart disease, cancer, stroke, or emphysema and having BMI values between 18.5 and 24.9 kg/m^2^, differences in mortality rates for current versus never smokers were 721.0 and 519.6 in men and women, respectively.

Table 4 shows the minimally adjusted and multivariable-adjusted HRs for death across categories of BMI and for continuous (per 5 kg/m^2^) values of BMI restricted to the BMI 40.0–59.9 kg/m^2^ range. In minimally adjusted models, we observed a sharp gradient in relative risk with higher values of BMI across the class III obesity levels that was consistent with a linear increase. The HRs from models of BMI categories were slightly, but not fully, attenuated with additional adjustment for race/ethnicity, education, alcohol intake, and physical activity level. Compared with adults with BMI in the 18.5–24.9 kg/m^2^ range, multivariable-adjusted HRs (95% CIs) for those with BMI of 40.0–44.9, 45.0–49.9, 50.0–54.9, and 55.0–59.9 kg/m^2^ were 2.25 (2.07–2.43), 3.32 (2.92–3.77), 3.48 (2.82–4.31), and 5.91 (4.24–8.24), respectively. This trend of increasing risk of death with increasing level of BMI was apparent in categorical and continuous models (HR per 5 kg/m^2^ = 1.40, 95% CI: 1.31–1.51) after restricting to the BMI 40.0–59.9 kg/m^2^ range.

**Table 4:** Hazard ratios and 95% confidence intervals for risk of death by BMI category.

Model Type	BMI (kg/m^2^) Category					Continuous BMI (per 5 kg/m^2^), Restricted to BMI 40.0–59.9 kg/m^2^
	18.5–24.9 (*n* = 304,011)	40.0–44.9 (*n* = 6,803)	45.0–49.9 (*n* = 1,978)	50.0–54.9 (*n* = 627)	55.0–59.9 (*n* = 156)	
Number of deaths	17,047	669	245	87	35	1,036
Minimally adjusted models^a^	1.00 (reference)	2.52 (2.33–2.72)	3.76 (3.32–4.27)	3.97 (3.21–4.90)	6.42 (4.61–8.95)	1.41 (1.31–1.52)
Multivariable-adjusted models^b^	1.00 (reference)	2.25 (2.07–2.43)	3.32 (2.92–3.77)	3.48 (2.82–4.31)	5.91 (4.24–8.24)	1.40 (1.31–1.51)
Alternate reference group models^b^^,^ c	—	1.00 (reference)	1.48 (1.28–1.72)	1.68 (1.34–2.10)	2.81 (1.99–3.97)	

a Models use attained age as the underlying time metric and were adjusted for sex and study.

b Models use attained age as the underlying time metric, and are adjusted for sex, race/ethnicity (white [all participants in the Swedish and Australian cohorts were coded as white], black, Asian/Pacific Islander, Hispanic, other/unknown), education (high school or less, post–high school, college, unknown), alcohol intake (grams of ethanol per day: 0, >0 to <10, ≥10, unknown), physical activity level (cohort-specific tertiles corresponding to low, medium, and high), and study.

c Using BMI = 40.0–44.9 kg/m^2^ as the reference group.

**Figure 1:**
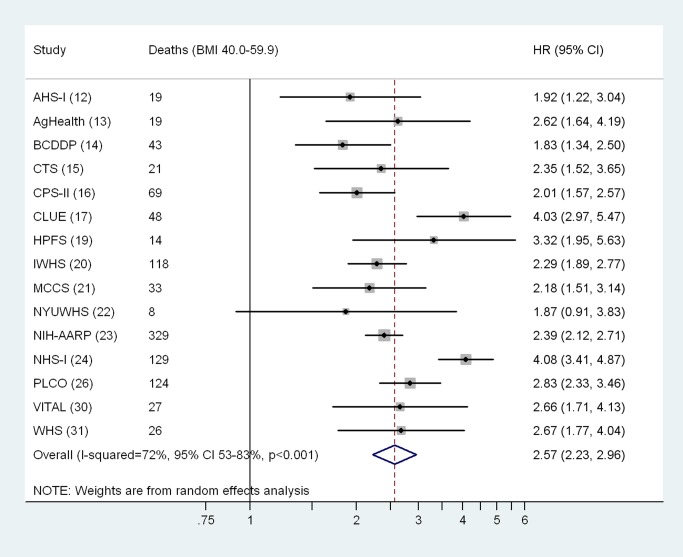
Cohort-specific and overall hazard ratios and 95% confidence intervals for BMI 40.0–59.9 kg/m^2^ versus 18.5–24.9 kg/m^2^. Results for cohorts with fewer than five deaths in the BMI 40.0–59.9 kg/m^2^ group are not shown. Cohort-specific models use attained age as the underlying time metric, and are adjusted for sex, race/ethnicity (white [all participants in the Swedish and Australian cohorts were coded as white], black, Asian/Pacific Islander, Hispanic, other/unknown), education (high school or less, post–high school, college, unknown), alcohol intake (grams of ethanol per day: 0, >0 to <10, ≥10, unknown), and physical activity level (cohort-specific tertiles corresponding to low, medium, and high). Random effects models were used to calculate overall (pooled) HRs and 95% CIs. The cohort acronyms are identified in Table 1, and references are given in parentheses

**Figure 2:**
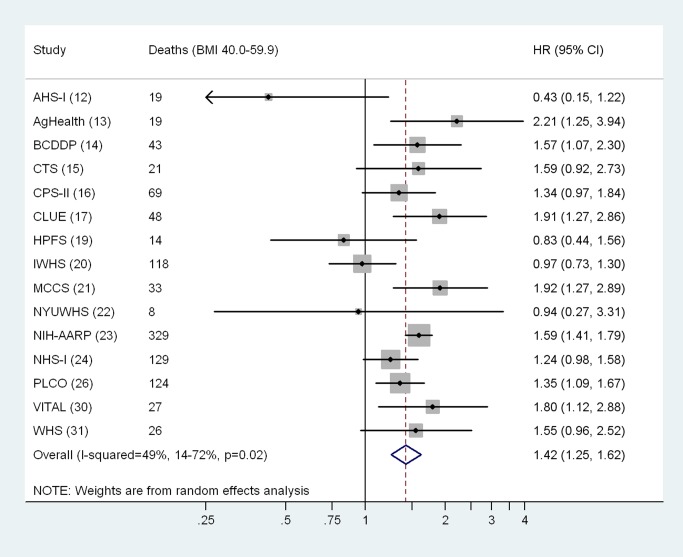
Cohort-specific and overall hazard ratios and 95% confidence intervals for continuous BMI (per 5 kg/m^2^). Analyses were restricted to participants with BMI 40.0–59.9 kg/m^2^. Results for cohorts with fewer than five deaths in the BMI 40.0–59.9 kg/m^2^ group are not shown. Cohort-specific models use attained age as the underlying time metric, and are adjusted for sex, race/ethnicity (white [all participants in the Swedish and Australian cohorts were coded as white], black, Asian/Pacific Islander, Hispanic, other/unknown), education (high school or less, post–high school, college, unknown), alcohol intake (grams of ethanol per day: 0, >0 to <10, ≥10, unknown), and physical activity level (cohort-specific tertiles corresponding to low, medium, and high). Random effects models were used to calculate overall (pooled) HRs and 95% CIs. The cohort acronyms are identified in Table 1, and references are given in parentheses.

Although the pooled HRs calculated from random effects meta-analysis models were similar to those based on the aggregate dataset (BMI 40.0–59.9 versus 18.5–24.9 kg/m^2^, HR = 2.57, 95% CI: 2.23–2.96; per 5 kg/m^2^ BMI, HR = 1.42, 95% CI: 1.25–1.62), the heterogeneity estimates for differences in the associations by cohort were statistically significant (Figures 1 and 2). We evaluated potential sources of heterogeneity in Table 5, which shows the multivariable-adjusted HRs conducted overall and by population subgroup. Overall, we observed a 2.57-fold (95% CI: 2.41–2.74) increased risk of death in the BMI 40.0–59.9 kg/m^2^ versus the 18.5–24.9 kg/m^2^ group, and the association increased linearly for BMI values above 40 kg/m^2^ (HR per 5 kg/m^2^ = 1.40, 95% CI: 1.31–1.51). As observed in studies of BMI across the entire range of values in relation to mortality [3], these associations were significantly modified by sex (*p*-interaction = 0.03), by geographic location (US versus non-US, *p*-interaction = 0.03), and follow-up time before and after age 65 y (*p*-interaction <0.001), with stronger associations observed for men (HR = 2.83, 95% CI: 2.45–3.26), for US cohorts (HR = 2.60, 95% CI: 2.44–2.78), and for follow-up before age 65 y (HR = 3.10, 95% CI: 2.77–3.47). The interaction by geographic location may have arisen due to chance, as there were only 37 deaths in the class III obesity group among non-US cohorts. No differences were observed for follow-up time occurring before versus after the year 2000. The HR for BMI 40.0–59.9 kg/m^2^ versus 18.5–24.9 kg/m^2^ did not change importantly after excluding the largest cohort, NIH-AARP, though the continuous HR for BMI values over 40 kg/m^2^ was slightly attenuated after this exclusion (HR = 1.32, 95% CI: 1.20–1.45). Exclusion of participants who reported a history of diabetes at baseline slightly attenuated the HR for BMI 40.0–59.9 kg/m^2^ versus 18.5–24.9 kg/m^2^ (HR = 2.27, 95% CI: 2.10–2.45). Exclusion of the first 2 y of follow-up did not appreciably change the HRs (<5% change). Although physical activity could confound or mediate these associations, removal of the physical activity variable also had little influence on the HRs (<5% change).

**Table 5:** Hazard ratios and 95% confidence intervals for risk of death according to BMI category (40.0–59.9 versus 18.5–24.9 kg/m^2^) or continuous values of BMI between 40.0 and 59.9 kg/m^2^ (per 5 kg/m^2^): subgroup analyses.

Subgroup	Category	Categorical BMI			Continuous BMI	
		BMI 18.5–24.9 kg/m^2^ (*n* = 304,011)	BMI 40.0–59.9 kg/m^2^ (*n* = 9,564)	*p*-Interaction*	Per 5 kg/m^2^ for BMI 40.0–59.9 kg/m^2^ (*n* = 9,564)	*p*-Interaction*
**Overall**	Number of deaths	17,047	1,036		1,036	
	HR (95% CI)^a^	1.00 (reference)	2.57 (2.41–2.74)		1.40 (1.31–1.51)	
**By sex**	**Men**					
	Number of deaths	5,404	212		212	
	HR (95% CI)^a^	1.00 (reference)	2.83 (2.45–3.26)		1.54 (1.32–1.81)	
	**Women**					
	Number of deaths	11,643	824		824	
	HR (95% CI)^a^	1.00 (reference)	2.53 (2.35–2.73)	0.03	1.37 (1.26–1.49)	0.01
**By attained age**	**Follow-up before age 65 y**					
	Number of deaths	3,830	368		368	
	HR (95% CI)^a^^,^ b	1.00 (reference)	3.10 (2.77–3.47)		1.59 (1.42–1.79)	
	**Follow-up after age 65 y**					
	Number of deaths	13,217	668		668	
	HR (95% CI)^a^^,^ c	1.00 (reference)	2.34 (2.16–2.53)	<0.001	1.30 (1.18–1.43)	<0.001
**By calendar year**	**Follow-up before 2000**					
	Number of deaths					
	HR (95% CI)^a^^,^ d	1.00 (reference)	2.50 (2.28–2.76)		1.35 (1.21–1.51)	
	**Follow-up after 2000**					
	Number of deaths					
	HR (95% CI)^a^^,^ e	1.00 (reference)	2.48 (2.27–2.71)	0.50	1.45 (1.32–1.60)	0.34
**By geography**	**US cohorts only**					
	Number of deaths	15,770	999		999	
	HR (95% CI)^a^	1.00 (reference)	2.60 (2.44–2.78)		1.39 (1.29–1.50)	
	**Non-US cohorts only**					
	Number of deaths	1,277	37		37	
	HR (95% CI)^a^	1.00 (reference)	1.88 (1.34–2.63)	0.03	1.71 (1.17–2.50)	0.04

*Calculated by including a cross-product term between the subgroup variable and BMI (40.0–59.9 versus 18.5–24.9 kg/m^2^); subgroup analyses for attained age and calendar year were stratified by follow-up period.

a Models use attained age as the underlying time metric, and are adjusted for sex, race/ethnicity (white [all participants in the Swedish and Australian cohorts were coded as white], black, Asian/Pacific Islander, Hispanic, other/unknown), education (high school or less, post–high school, college, unknown), alcohol intake (grams of ethanol per day: 0, >0 to <10, ≥10, unknown), physical activity level (cohort-specific tertiles corresponding to low, medium, and high), and study.

b Models excluded participants who entered the study at or after age 65 y; follow-up time was censored at age at study exit (due to loss to follow-up, death, or administrative end date) or age 65 y, whichever came first.

c Models excluded participants who exited the study at or before age 65 y; follow-up began at age 65 y for those who entered the study before age 65 y.

d Models excluded participants who entered the study on or after the year 2000; follow-up time was censored at study exit (due to loss to follow-up, death, or administrative end date) or the year 2000, whichever came first.

e Models excluded participants who exited the study on or before the year 2000; follow-up began in the year 2000 for those who entered the study before the year 2000.

Multivariable-adjusted HRs for select major causes of death for BMI 40.0–59.9 kg/m^2^ compared with 18.5–24.9 kg/m^2^ are shown in Table 6. HRs were significantly elevated for deaths due to heart disease, malignant neoplasms, cerebrovascular disease, diabetes mellitus, nephritis/nephritic syndrome/nephrosis, chronic lower respiratory disease, influenza/pneumonia, accidents, and septicemia, ranging from 1.69 for deaths due to malignant neoplasms to 9.57 for deaths due to nephritis/nephrotic syndrome/nephrosis. Additional adjustment for self-reported history of diabetes and hypertension attenuated the HRs for all specific causes of death apart from malignant neoplasms, though all remained statistically significant. Within the BMI 40.0–59.9 kg/m^2^ range, greater BMI values were linearly associated with increasing HRs for all of these major causes of death apart from cerebrovascular disease, accidents, and septicemia; additional adjustment for history of diabetes and hypertension had little influence on these continuous HRs.

**Table 6:** Hazard ratios and 95% confidence intervals for select major causes of death by BMI category.

Model Type	Categorical BMI		Continuous BMI (per 5 kg/m^2^) for BMI 40.0–59.9 kg/m^2^ (*n* = 9,564)
	18.5–24.9 kg/m^2^ (*n* = 304,011)	40.0–59.9 kg/m^2^ (*n* = 9,564)	
**Total deaths**			
Number of deaths	17,047	1,036	1,036
HR (95% CI) for multivariable model^a^	1.00 (reference)	2.57 (2.41–2.74)	1.40 (1.31–1.51)
HR (95% CI) for additionally adjusted model^b^	1.00 (reference)	2.10 (1.96–2.25)	1.39 (1.29–1.49)
**Heart disease**			
Number of deaths	3,813	345	345
HR (95% CI) for multivariable model^a^	1.00 (reference)	4.03 (3.83–4.84)	1.30 (1.14–1.48)
HR (95% CI) for additionally adjusted model^b^	1.00 (reference)	3.26 (2.89–3.68)	1.28 (1.13–1.46)
**Malignant neoplasms**			
Number of deaths	7,161	294	294
HR (95% CI) for multivariable model^a^	1.00 (reference)	1.69 (1.50–1.90)	1.27 (1.10–1.47)
HR (95% CI) for additionally adjusted model^b^	1.00 (reference)	1.63 (1.45–1.84)	1.28 (1.10–1.47)
**Cerebrovascular disease**			
Number of deaths	1,599	52	52
HR (95% CI) for multivariable model^a^	1.00 (reference)	1.67 (1.26–2.22)	0.73 (0.46–1.16)
HR (95% CI) for additionally adjusted model^b^	1.00 (reference)	1.35 (1.01–1.80)	0.69 (0.43–1.10)
**Diabetes mellitus**			
Number of deaths	235	61	61
HR (95% CI) for multivariable model^a^	1.00 (reference)	8.82 (6.46–12.05)	1.55 (1.16–2.06)
HR (95% CI) for additionally adjusted model^b^	1.00 (reference)	2.24 (1.61–3.11)	1.50 (1.13–1.99)
**Nephritis, nephrotic syndrome, and nephrosis**			
Number of deaths	149	36	36
HR (95% CI) for multivariable model^a^	1.00 (reference)	9.57 (6.41–14.27)	2.14 (1.55–2.97)
HR (95% CI) for additionally adjusted model^b^	1.00 (reference)	6.27 (4.11–9.57)	2.06 (1.48–2.85)
**Chronic lower respiratory disease**			
Number of deaths	255	17	17
HR (95% CI) for multivariable model^a^	1.00 (reference)	2.33 (1.39–3.88)	2.00 (1.21–3.31)
HR (95% CI) for additionally adjusted model^b^	1.00 (reference)	2.15 (1.28–3.62)	2.02 (1.21–3.36)
**Influenza and pneumonia**			
Number of deaths	508	18	18
HR (95% CI) for multivariable model^a^	1.00 (reference)	1.82 (1.13–2.95)	1.76 (1.09–2.85)
HR (95% CI) for additionally adjusted model^b^	1.00 (reference)	1.45 (0.89–2.37)	1.75 (1.07–2.86)
**Accidents (unintentional injuries)**			
Number of deaths	771	31	31
HR (95% CI) for multivariable model^a^	1.00 (reference)	1.76 (1.22–2.53)	1.16 (0.74–1.82)
HR (95% CI) for additionally adjusted model^b^	1.00 (reference)	1.61 (1.10–2.34)	1.15 (0.73–1.79)
**Septicemia**			
Number of deaths	139	14	14
HR (95% CI) for multivariable model^a^	1.00 (reference)	3.95 (2.21–7.08)	1.32 (0.68–2.55)
HR (95% CI) for additionally adjusted model^b^	1.00 (reference)	3.10 (1.69–5.67)	1.30 (0.67–2.51)

a Models use attained age as the underlying time metric, and are adjusted for sex, race/ethnicity (white [all participants in the Swedish and Australian cohorts were coded as white], black, Asian/Pacific Islander, Hispanic, other/unknown), education (high school or less, post–high school, college, unknown), alcohol intake (grams of ethanol per day: 0, >0 to <10, ≥10, unknown), physical activity level (cohort-specific tertiles corresponding to low, medium, and high), and study.

b Additionally adjusted for history of diabetes (ever diagnosed, never diagnosed, or unknown) and hypertension (ever diagnosed, never diagnosed, or unknown).

doi:10.1371/journal.pmed.1001673.t006

We estimated that BMI categories of 40–44.9, 45–49.9, 50–54.9, and 55–59.9 kg/m^2^ were associated with an estimated 6.5 (95% CI: 5.7–7.3), 8.9 (95% CI: 7.4–10.4), 9.8 (95% CI: 7.4–12.2), and 13.7 (95% CI: 10.5–16.9) y of life lost (Table 7). These estimates (95% CIs) were 6.5 (5.6–7.4), 10.8 (9.3–12.3), 10.6 (7.6–13.6), and 17.0 (12.9–21.1) in men and 6.9 (6.1–7.7), 9.1 (7.9–10.3), 10.3 (8.3–12.3), and 13.1 (9.0–17.2) in women. By comparison, in the subset of participants in the pooled dataset without heart disease, cancer, stroke, or emphysema and having BMI values between 18.5 and 24.9 kg/m^2^, current versus never cigarette smoking was associated with an estimated 8.9 y of life lost (95% CI: 8.6–9.3).

**Table 7:** Years of life lost and 95% confidence intervals by BMI category.

Group	BMI (kg/m^2^)				
	18.5–24.9 (*n* = 304,011)	40.0–44.9 (*n* = 6,803)	45.0–49.9 (*n* = 1,978)	50.0–54.9 (*n* = 627)	55.0–59.9 (*n* = 156)
Total	Reference	6.5 (5.7–7.3)	8.9 (7.4–10.4)	9.8 (7.4–12.2)	13.7 (10.5–16.9)
Men	Reference	6.5 (5.6–7.4)	10.8 (9.3–12.3)	10.6 (7.6–13.6)	17.0 (12.9–21.1)
Women	Reference	6.9 (6.1–7.7)	9.1 (7.9–10.3)	10.3 (8.3–12.3)	13.1 (9.0–17.2)

Data are years of life lost (95% CI). Based on proportional hazards regression models using attained age as the underlying time metric and adjusted for sex, race/ethnicity (white [all participants in the Swedish and Australian cohorts were coded as white], black, Asian/Pacific Islander, Hispanic, other/unknown), education (high school or less, post–high school, college, unknown), alcohol intake (grams per day: 0, >0 to <10, ≥10, unknown), physical activity level (cohort-specific tertiles corresponding to low, medium, and high), and study.

## Discussion

To our knowledge, this is the largest study to date on the association between class III obesity and mortality, and the first to show that class III obesity is associated with excess rates of total mortality and mortality due to a wide range of causes, particularly heart disease, cancer, and diabetes, and that the risk of death overall and from these specific causes continues to rise with increasing values of BMI. The accumulating excess risk resulted in major reductions in life expectancy after the age of 40 y that were comparable to those of cigarette smoking.

The estimated 6.5 to 13.7 y of life lost for BMI values between 40 and 59 kg/m^2^ versus 18.5–24.9 kg/m^2^ were in line with those of a previous pooled analysis that found that individuals with BMI values of 35–50 kg/m^2^ had a median 8–10 fewer years of life than those with BMI 22.5–24.9 kg/m^2^ [2]. Our study further demonstrates that the expected number of years of life lost continued to increase for BMI values beyond 50 kg/m^2^, at which point the loss in life expectancy (9.8 y) exceeded that observed for current versus never smoking (8.9 y) in this study. These results have great relevance to the current era, during which class III obesity rates have increased dramatically at the same time that smoking rates have declined. That we observed a 7.2-y decrease in life expectancy from BMI 40.0–44.9 to 55.0–59.9 kg/m^2^ suggests that otherwise healthy, non-smoking adults having BMI values within the class III obesity range may considerably expand their life expectancy by avoiding additional weight gain.

We observed significant differences in the relative risks for class III obesity and total mortality by cohort, which may reflect differences in the ages, sex distribution, and geographical location of the participants, as these factors were shown to be significant effect modifiers in our study. Similar to observations from previous studies based on lower values of BMI [3], these results suggest that the risks associated with class III obesity may be even greater for men than women, and for younger compared to older adults. These results could be considered in the development of public health interventions aimed at reducing health risks associated with extreme obesity, particularly those that are targeted toward susceptible populations.

The clear finding of excess risk for nearly every major cause of death suggests that excess weight can have a wide range of physiological effects. In particular, the results of our study suggest that diabetes and hypertension are common mechanisms that might explain the majority of the excess mortality burden in the class III obesity group. Most of this excess was attributable to heart disease, diabetes, cancer, and kidney and liver diseases, and risks of death due to these causes were largely attenuated with additional adjustment for diabetes and hypertension. Obesity is strongly associated with metabolic abnormalities, including insulin resistance, type 2 diabetes, hypertension, and dyslipidemia, mediated in part by the chronic inflammatory state induced by the secretion of adipocytokines, such as angiotensinogen, transforming growth factor–beta, tumor necrosis factor–alpha, and interleukin-six [42]–[45]. The severity of these conditions has been shown to increase with increasing BMI [44] and improve after weight loss either due to lifestyle modification or bariatric surgery [46]–[49]. Diabetes, hypertension, and other obesity-related aspects of the metabolic syndrome have been shown to play an important role in the development of heart disease, chronic kidney disease, and end-stage renal disease [42],[45], among other major causes of death observed to be elevated in the class III obesity group. Thus, these metabolic conditions could be effect targets in efforts to reduce the burden of morbidity and mortality in individuals with extreme obesity. Individuals with extreme obesity may experience other physiological impairments that could explain the higher rates of death from other causes. Specifically, deaths due to chronic lower respiratory disease were shown in this and the much smaller National Health and Nutrition Examination Survey III study to be elevated at extreme levels of BMI due to reduced lung capacity and airway obstruction [50]. It is possible that excess risks associated with class III obesity may decline as treatments for these co-morbid conditions improve.

An important strength of this pooling study is its large size. With sufficient numbers, we were able to quantify the risks of total and cause-specific deaths associated with class III obesity with greater precision than has been possible in previous studies. Furthermore, our results were based on a population of never smokers without prevalent disease, thereby reducing the potential for confounding due to major preexisting illness and smoking. Our results were also based on original, as opposed to published, data, which allowed for standardization of the variables and statistical methods across studies, and thereby reduced the potential for methodological heterogeneity between studies.

Some limitations of this study include the use of mostly self-reported, as opposed to measured, height and weight. Objectively measured values are ideal, but epidemiologic studies have reported high correlations between self-reported and measured height and weight [51],[52]. Nonetheless, misclassification across BMI categories due to self-reported height and weight has been shown to yield stronger relative risks than those based on measured values [53]. This potential source of bias may have inflated our relative risk estimates, although this effect is likely to have been modest [3]. Another limitation is that height and weight were ascertained only once at the beginning of follow-up, which precluded us from examining risks associated with weight change. Although BMI is the most commonly used indicator of adiposity in epidemiologic studies, it does not directly measure the amount of adipose tissue and cannot distinguish between fat and lean body mass [54],[55]. Nonetheless, BMI has been shown to be just as strongly correlated with obesity-related metabolic indicators as more accurate methods for measuring total fat mass and body fat percent, such as dual-energy X-ray absorptiometry [55]. BMI also does not provide information on central versus peripheral body fat distribution, each posing different risks in relation to the metabolic syndrome and related chronic diseases [56]. Studies that combine BMI with measures of central adiposity may yield more accurate estimates of disease risks associated with high levels BMI [57],[58]. For instance, in a pooled analysis based on data from the same 20 cohort studies, waist circumference was positively associated with all-cause mortality, even among individuals at the highest range of BMI (35.0–49.9 kg/m^2^) [58]. There has been some concern about the accuracy of cause of death reports [59], which may be differential by BMI level. The excess deaths due to both diabetes mellitus and overweight and obesity that we observed in the class III obesity group may reflect greater difficulty in identifying the primary cause of death among individuals with multiple co-morbid conditions. Our results may not be generalizable to all populations, as the pooled dataset comprised mostly non-Hispanic white individuals from affluent countries (the majority being from the US). Also, our estimates rely on prospective cohort studies, whose volunteer participants typically are healthier than those in the general population.

In this large pooled analysis of 20 prospective studies, participants with BMI in the class III obesity range (40.0–59.9 kg/m^2^) experienced substantially higher rates of death compared with those in the normal BMI range (18.5–24.9 kg/m^2^), with most of the excess due to deaths from heart disease, cancer, and diabetes. These higher rates appear to be largely attributable to metabolic abnormalities associated with excess adiposity, including diabetes and hypertension. We found that the reduction in life expectancy associated with class III obesity was similar to (and, for BMI values above 50 kg/m^2^, even greater than) that observed for current smoking. If current global trends in obesity continue, we must expect to see substantially increased rates of mortality due to these major causes of death, as well as rising health-care costs. These results underscore the need to develop more effective interventions to combat this growing public health problem.

## Abbreviations

BMI: body mass index
HR: hazard ratio
NIH-AARP: National Institutes of Health–American Association of Retired Persons Diet and Health Study
